# Differential Elimination of Anti-Thymocyte Globulin of Fresenius and Genzyme Impacts T-Cell Reconstitution After Hematopoietic Stem Cell Transplantation

**DOI:** 10.3389/fimmu.2019.00315

**Published:** 2019-03-06

**Authors:** Lisa V. E. Oostenbrink, Cornelia M. Jol-van der Zijde, Katrine Kielsen, Anja M. Jansen-Hoogendijk, Marianne Ifversen, Klaus G. Müller, Arjan C. Lankester, Astrid G. S. van Halteren, Robbert G. M. Bredius, Marco W. Schilham, Maarten J. D. van Tol

**Affiliations:** ^1^Department of Pediatrics, Leiden University Medical Center, Leiden, Netherlands; ^2^Institute for Inflammation Research, Copenhagen University Hospital Rigshospitalet, Copenhagen, Denmark; ^3^Department of Paediatrics and Adolescent Medicine, Copenhagen University Hospital Rigshospitalet, Copenhagen, Denmark

**Keywords:** ATG, Genzyme, Fresenius, serotherapy, pediatrics, acute GvHD

## Abstract

Anti-thymocyte globulin (ATG) is a lymphocyte depleting agent applied in hematopoietic stem cell transplantation (HSCT) to prevent rejection and Graft-vs.-Host Disease (GvHD). In this study, we compared two rabbit ATG products, ATG-Genzyme (ATG-GENZ), and ATG-Fresenius (ATG-FRES), with respect to dosing, clearance of the active lymphocyte binding component, post-HSCT immune reconstitution and clinical outcome. Fifty-eigth pediatric acute leukemia patients (*n* = 42 ATG-GENZ, *n* = 16 ATG-FRES), who received a non-depleted bone marrow or peripheral blood stem cell graft from an unrelated donor were included. ATG-GENZ was given at a dosage of 6–10 mg/kg; ATG-FRES at 45–60 mg/kg. The active component of ATG from both products was cleared at different rates. Within the ATG-FRES dose range no differences were found in clearance of active ATG or T-cell re-appearance. However, the high dosage of ATG-GENZ (10 mg/kg), in contrast to the low dosage (6–8 mg/kg), correlated with prolonged persistence of active ATG and delayed T-cell reconstitution. Occurrence of serious acute GvHD (grade III–IV) was highest in the ATG-GENZ-low dosage group. These results imply that dosing of ATG-GENZ is more critical than dosing of ATG-FRES due to the difference in clearance of active ATG. This should be taken into account when designing clinical protocols.

## Introduction

Serotherapy with lymphocyte depleting antibodies, particularly anti-thymocyte globulin (ATG) and alemtuzumab, is frequently applied as part of the conditioning regimen in allogeneic hematopoietic stem cell transplantation (HSCT). Its major aim is to reduce the risk of Graft-vs.-Host Disease (GvHD) and graft rejection. ATG targets cells of the immune system, such as T-cells (1–3), through various mechanisms (3, 4). ATG products are polyclonal IgG antibodies, raised in horses or rabbits by immunization with human thymocytes (horse: ATGAM®, Pfizer, New York, NY, USA; rabbit ATG-Genzyme, ATG-GENZ, Thymoglobulin®, Sanofi Genzyme, Cambridge, MA, USA), or with a Jurkat T-cell line (rabbit ATG-Fresenius, ATG-FRES, recently re-named as anti-human T-lymphocyte immunoglobulin ATLG, Grafalon®, Neovii Biotech, Rapperswil, Switzerland). Both ATG-GENZ and ATG-FRES not only target T-cells but also, amongst others, neutrophils, monocytes, NK-, B-cells and non-immune cells like endothelial cells (5). However, due to the differences in manufacturing procedures, both products contain different specificities and quantity of antibodies (5, 6). As a consequence, the lymphodepleting potency of the two rabbit ATG products, ATG-GENZ and ATG-FRES, differs, which is reflected in the historically recommended total dosage for use in pre-HSCT conditioning, i.e., in children 10 mg/kg for ATG-GENZ and 60 mg/kg for ATG-FRES (7–10). The preferential use of either ATG product depends on (inter)national guidelines, disease protocol or the physicians choice (5, 11, 12). Active ATG is defined as the fraction of ATG that is capable of binding to lymphocytes. Its presence in serum or plasma can be determined by flow cytometry (13). Active ATG makes up only < 10% of the total rabbit IgG (total ATG). The clearance of active ATG has, in contrast to clearance of total rabbit IgG, a major impact on immune recovery and clinical outcome post-HSCT (7–9, 11, 14–16). The level of active ATG of 1 AU/mL is considered the threshold above which T-cell reconstitution is never observed (15). Therefore, the optimal approach for monitoring ATG is regular measurement of active ATG levels, rather than total ATG (rabbit IgG).

In several recent reports, ATG-GENZ and ATG-FRES were compared as part of the conditioning regimen pre-HSCT (10, 17–21) or as induction treatment in solid organ transplant recipients (22). With one exception (10), these studies suggest that immune recovery is delayed after usage of ATG-GENZ compared to ATG-FRES with controversial data regarding the impact on clinical outcome (18–22). However, reports comparing active ATG-GENZ and ATG-FRES are lacking. In addition, in various international protocols the dosages of ATG-GENZ as well as ATG-FRES have recently been reduced, with the aim to promote more rapid immune recovery, Graft-vs.-Leukemia (GvL) and protection against infections (23–27). Therefore, the aim of the present study was to compare the clearance of the active components after dose reduction of ATG-GENZ and ATG-FRES post-HSCT and to investigate the impact on immune recovery, viral infections and the incidence of acute and chronic GvHD in a homogeneous cohort of children receiving allogeneic HSCT.

## Patients and methods

### Patients

Children treated between 2010 and 2016 for acute lymphoblastic (ALL) or acute myeloid leukemia (AML) with an allogeneic HSCT and receiving ATG as part of the pre-HSCT myeloablative conditioning regimen were eligible for this study. Patients were transplanted in the Leiden University Medical Center (*n* = 38) or the Copenhagen University Hospital Rigshospitalet (*n* = 20) with a non-depleted bone marrow (BM) or peripheral blood stem cell (PBSC) graft from an unrelated donor. All patients and donors were genotyped using high resolution typing for HLA class I and II alleles (10 antigens: -A, -B, -C, -DR^*^B1, -DQ^*^B1). HLA-matched donors were defined as 10 out of 10 matched. Standard care consisted of protective isolation and infection prophylaxis, both performed in accordance with institutional guidelines. No cytomegalovirus (CMV), Epstein-Barr virus (EBV) or human adenovirus (HAdV) prophylaxis was used. Surveillance of viral reactivation was performed during the period after HSCT, up to time of T-cell recovery, through measuring plasma DNA load twice a week. GvHD prophylaxis consisted of cyclosporine A (CsA) and methotrexate (MTX) (*n* = 52), tacrolimus in case of CsA intolerance (*n* = 1) or a combination of CsA/MTX and mycophenolate mofetil (MMF) (*n* = 5). In the study cohort of 58 patients (Table 1), ATG-GENZ was given at the historical dosage of 10 mg/kg (*n* = 24) or the lowered dosage of 6–8 mg/kg (*n* = 18) and ATG-FRES at 60 mg/kg (*n* = 9) or 45 mg/kg (*n* = 7) (Supplementary Table 1). Immune cell recovery up to 1 year, incidence of viral infections up to 180 days, occurrence of acute GvHD up to 100 days post-HSCT, chronic GvHD up to 1 year and cumulative incidence of relapse and overall survival up to 5 years were investigated as outcome parameters. Patients were excluded for further analysis of immune recovery when late graft rejection and early leukemia relapse occurred. Immune reconstitution of patients who received ATG was compared with data obtained from 22 children transplanted for acute leukemia with an HLA identical donor, without serotherapy in the conditioning (Leiden *n* = 13, Copenhagen *n* = 9). Institutional review boards approved this study (Leiden, P01.028; Copenhagen, ATG H-1-2010-009/H-7-2014-016), and informed consent was obtained from all patients and/or their legal guardians in accordance with the declaration of Helsinki.

**Table 1:** Patient characteristics ATG-Genzyme and ATG-Fresenius groups.

	**ATG Genzyme**	**ATG Fresenius**	***p*-value**
Number of patients	42	16	
**TRANSPLANT CENTER**, ***n*** **(%)**			
Leiden	38 (90)		
Copenhagen	4 (10)	16 (100)	
Patient age, years; median (range)	9 (1–18)	6 (1–17)	0.248
**DIAGNOSIS**, ***n*** **(%)**			
Acute lymphoblastic leukemia	17 (40)	16 (100)	<0.001
Acute myeloid leukemia	25 (60)	–	
**DONOR**, ***n*** **(%)**			
HLA match 10/10	30 (71)	13 (81)	0.52
HLA match 8–9/10	12 (29)	3 (19)	
**STEM CELL SOURCE**, ***n*** **(%)**			
Bone marrow	34 (81)	14 (87)	0.71
Peripheral blood	8 (19)	2 (13)	
**TOTAL NC DOSE, 10**^**8**^**/kg BW; median (range)**			
Bone marrow graft	2.8 (0.6–9.6)	5.5 (2.3–7.9)	0.02
Peripheral blood graft	8.3 (5.0–16.4)	16.1^*^	–
**SEROTHERAPY ATG, mean (range)**			
Genzyme; mg/kg BW	8.7 (6.0–10.5)	–	–
Fresenius; mg/kg BW	–	53 (45–60)	
**SEROTHERAPY PARAMETERS, median (range)**			
Start serotherapy, day pre-HSCT	−6 (−8 to −3)	−4 (−7 to −3)	<0.001
Days ATG infusion	3 to 4	3	<0.001
Bodyweight, kg	35 (9 to 84)	25 (9 to 69)	0.141
Lymphocytes pre-ATG, × 10^9^/L	0.2 (0.01 to 1.2)	0.05 (0.01 to 0.7)	0.10
**CONDITIONING REGIMEN**, ***n*** **(%)**			
Chemotherapy + TBI	6 (14)	9 (56)	0.002
Chemotherapy	36 (86)	7 (44)	
**GvHD PROPHYLAXIS**, ***n*** **(%)**			
CSA + MTX	36 (86)	16 (100)	0.04
MTX + tacrolimus	1 (2)		
CSA + MTX + MMF	5 (12)		
**PATIENTS AT RISK**^†^, ***n*** (**%**)			
CMV	29 (69)	14 (87)	0.19
EBV	41 (98)	13 (100)^*^	1.00

*BW, body weight; CMV, cytomegalovirus; CsA, cyclosporine A; EBV, Epstein-Barr virus; MTX, methotrexate; MMF, mycofenolate mofetil; NC, nucleated cells; TBI, total body irradiation*.

* *Data of 1 patient (NC dose) and data of the EBV status of 3 donor-patients couples pre-conditioning were lacking*.

† *CMV and EBV seropositive patients and seronegative patients with a seropositive donor*.

### Rabbit IgG and Active ATG

ATG levels were measured in sera/plasma samples taken before start of the conditioning therapy, at the day of graft infusion and at 1, 2, 3, 4, 8, and 13 weeks thereafter. An additional sample was collected from Leiden patients at 6 weeks post-HSCT. Samples were collected as clotted or EDTA-anti-coagulated blood, centrifuged shortly after collection and stored at −20 or −80°C.

Concentrations of rabbit IgG (total ATG) were measured by ELISA (28). Microtiter plates were coated with goat anti-rabbit IgG adsorbed for human IgG (Jackson Europe, Suffolk, UK). After blocking and washing, a standard curve of ATG-GENZ (Thymoglobulin, range 4–250 ng/mL) and diluted patients sera (start at least 1:100, 2-fold dilutions) were incubated for 1 h, followed by washing and incubation with alkaline phosphatase-conjugated goat anti-rabbit IgG adsorbed for human IgG (Jackson Europe).

In a modification of the method described earlier, active ATG, the fraction of the product capable of binding to cells, was measured in serum or plasma samples using a quantitative flow cytometry assay (29). In short, HUT-78 T-cells were incubated with 4-fold dilutions of patient's serum/plasma, starting with a dilution of 1:4. To construct a reference curve, HUT-cells were incubated with known concentrations of ATG (Thymoglobulin, Genzyme). Active ATG is expressed in arbitrary units (AU). Five mg ATG-GENZ was arbitrarily set to contain 5,000 AU of active ATG. The range of the standard curve (2-fold dilutions in triplicate), is from 0.005 to 5 AU/mL. For validation of the method, the ATG-GENZ reference curve was compared with a reference curve constructed with similar rabbit IgG concentrations of ATG-FRES (Supplementary Figure 1A). After exposure to sample and standard dilutions the cells were washed and incubated with Alexa Fluor 647 labeled goat anti-rabbit IgG (Life Technologies, Carlsbad, CA, USA). Finally, cells were washed and analyzed by flow cytometry on a FACS Scan (Becton Dickinson Biosciences, Franklin Lakes, NJ, USA). Mean fluorescence intensities obtained at the different standard dilutions were plotted against the active ATG concentrations. The lower limit of quantification for active ATG was 0.1 AU/mL. Since HUT-78 cells do not express all of the antigens expressed on normal T-lymphocytes, we validated our data by comparing the binding of active ATG in patients samples obtained at different time points after ATG-GENZ or ATG-FRES infusion to HUT-78 cells with the binding to the T-cell fraction from peripheral blood mononuclear cells (PBMC) (Supplementary Figure 1B).

In order to investigate the impact of active ATG exposure on immune reconstitution, the day post-HSCT that the active ATG level fell below 1 AU/mL was calculated. Above this level, T-cells did not display numbers ≥ 100 cells/μL, in any of the patients (15, 28).

All patients were screened for the presence of IgM, IgA, and IgG anti-rabbit IgG (IgM, IgA, and IgG anti-ATG) antibodies at 30 and 60 days post-HSCT using an ELISA as described before (29). When anti-rabbit antibodies were detected, earlier post-HSCT samples were analyzed as well. In 6 patients (all ATG-GENZ), IgG antibodies against rabbit IgG were detected. These patients were excluded from further analysis of rabbit IgG levels from the time of detection of these antibodies. Of these 6 ATG-GENZ patients with anti-rabbit IgG antibodies, only one developed these antibodies when active ATG was still present. Therefore, this patient was excluded from the day of anti-rabbit IgG appearance (day 18 post-HSCT) onwards for the active ATG analysis. All 6 patients were included in the analysis of immune cell recovery and clinical outcome analysis, since all of them had an active ATG concentration <1.0 AU/mL at the time anti-rabbit IgG became detectable. Because 1 AU/ml is the critical threshold allowing T-cell recovery, the occurrence of anti-ATG antibodies in these patients is considered not biologically relevant.

### Immune Cell Recovery

Engraftment was defined as the first day of 2 consecutive measurements at which the absolute neutrophil count was above 0.5 × 10^9^/L in the absence of neutrophil infusions. Absolute lymphocyte counts (leukocyte count and differential) and lymphocyte subpopulations (CD3^+^ T-cells, CD3^+^CD4^+^, and CD3^+^CD8^+^ T-cell subsets, CD3^−^CD56^+^/CD16^+/−^ NK-cells and CD19+/CD20+ B-cells) in PBMC were measured in fresh material as part of routine clinical evaluation. In all patients PBMC were investigated at 1, 2, 3, 6, and 12 months post-HSCT by immunostaining and flow cytometry (Leiden: FACS Calibur II, Becton Dickinson Biosciences, Franklin Lakes, NJ, USA; Copenhagen: FC500 flow cytometer, Beckman Coulter, Brea, CA, USA). Data were analyzed using BD CellQuest and FACS Diva software.

### Clinical Outcome Parameters

Incidences of acute and chronic GvHD were classified using the Glucksberg and Shulman criteria, respectively (30, 31). Acute GvHD referred to all grades (I-IV), whereas severe acute GvHD was defined as grade III-IV. Chronic GvHD referred to limited/extended. Data were censored for relapse and second transplant. CMV and EBV infections/reactivations were defined as two consecutive log serum viral DNA loads of at least 3.0 determined by real time quantitative (RQ) PCR in the first 100 days after transplantation (32, 33). Pretransplant CMV and EBV serostatus of patient and donor were determined for all HSCT couples and only CMV and EBV seropositive patients and seronegative patients with a seropositive donor (at risk patients) were included in CMV and EBV infection/reactivation analyses. Overall survival (OS) was defined as time to death, regardless of cause, or last follow-up (censoring), event-free survival (EFS) referred to the time to disease reoccurrence, retransplantation, death or last follow-up.

### Statistical Analysis

All variables and outcome parameters were compared between the ATG-GENZ and ATG-FRES groups. Differences in patient characteristics among the treatment groups (Table 1) were compared using Mann-Whitney rank tests for continuous data, chi-square tests for categorical data and Fisher's exact tests for binomial data (SPSS 22). After dividing the ATG-GENZ group in ATG-GENZ-high and ATG-GENZ-low, the analyses to compare the ATG-GENZ subgroups and the ATG-FRES group were performed by Kruskal-Wallis or chi-square tests. Comparison of immune recovery at different time points was done on log transformed data by *T*-tests or ANOVA. The cumulative incidence of acute GvHD, viral infections/reactivations and the recovery of immune cells were calculated using the Kaplan-Meier method. The probabilities of acute GvHD, chronic GvHD, relapse, viral reactivations and the recovery of immune cells were compared with log-rank tests. Graphs were made in Prism Graphpad 7.06 (Graphpad Software, La Jolla, CA, USA).

## Results

### Clearance of Rabbit IgG (Total ATG)

**Figure 1:**
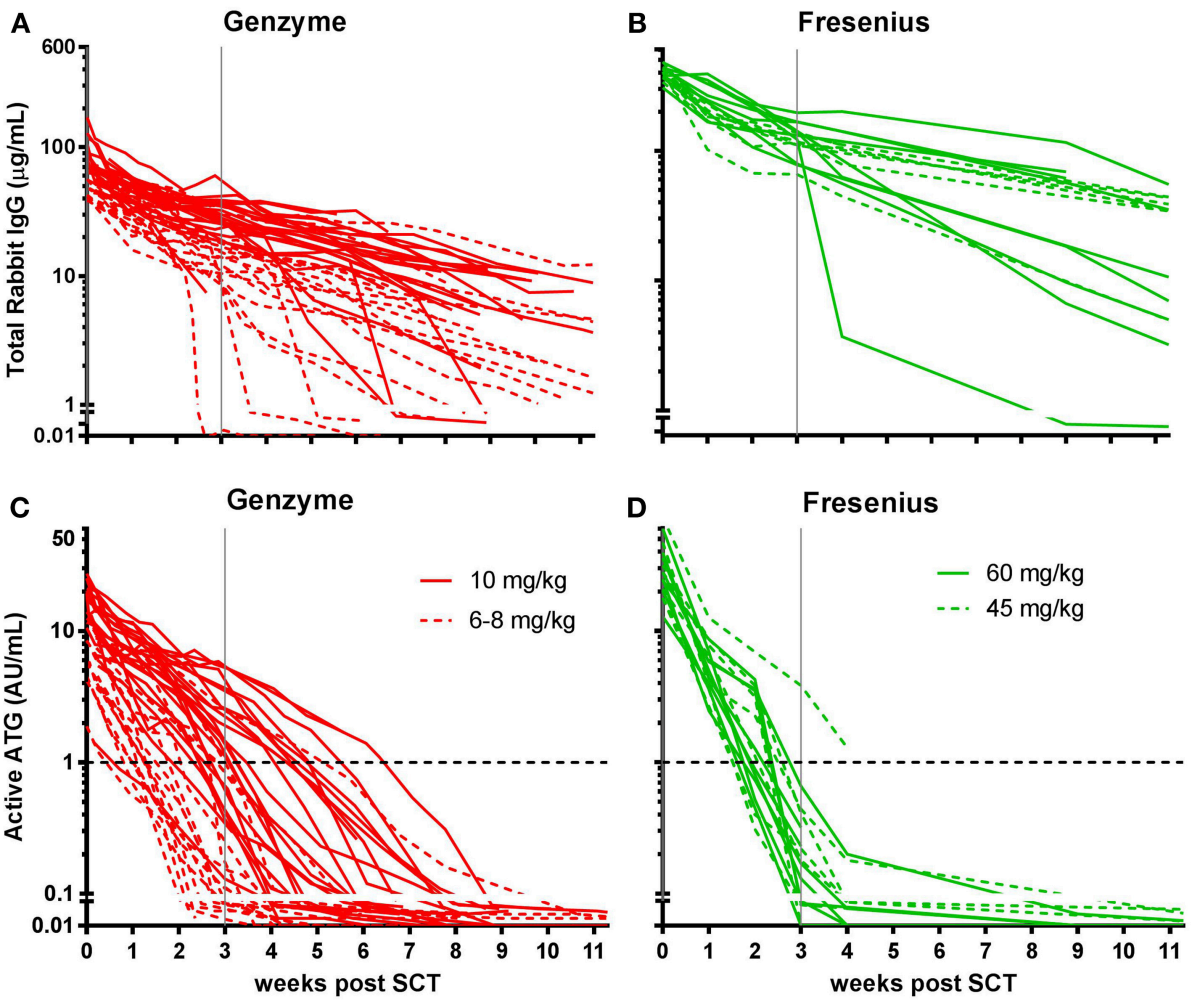
Serum/plasma concentration of total Rabbit IgG (total ATG) and active ATG in ATG-GENZ (Genzyme, red) or ATG-FRES (Fresenius, green) treated patients. Total Rabbit IgG (μg/mL) of ATG-FRES treated patients **(B)** was higher in comparison with ATG-GENZ treated patients **(A)**, because of the higher dose of ATG-FRES given, but clearance was comparable for both brands. Active ATG levels (AU/mL) of ATG-FRES treated patients **(D)**, receiving 60 mg/kg (solid lines; *n* = 9) or 45 mg/kg (dashed lines; *n* = 7), decreased fast and was only in 1 out of 16 patients (6%) above 1 AU/mL at 3 weeks post-HSCT. ATG-GENZ treated patients **(C)** receiving 10 mg/kg (solid lines; *n* = 24) or 6–8 mg/kg (dashed lines; *n* = 18) showed more variance in clearance. At 3 weeks post-HSCT 15 out of 24 patients (63%) receiving 10 mg/kg had active ATG levels above 1 AU/mL vs. only 2 out of 18 patients (11%) receiving 6–8 mg/kg. The horizontal dashed line at 1 AU/mL marks the highest serum/plasma concentration at which T-cells can reappear in the blood. The vertical solid gray line is the expected time of neutrophil engraftment. Of note, in the 6 patients receiving ATG-GENZ and showing a steep decline of rabbit ATG IgG antibodies against rabbit IgG were detected.

**Figure 2:**
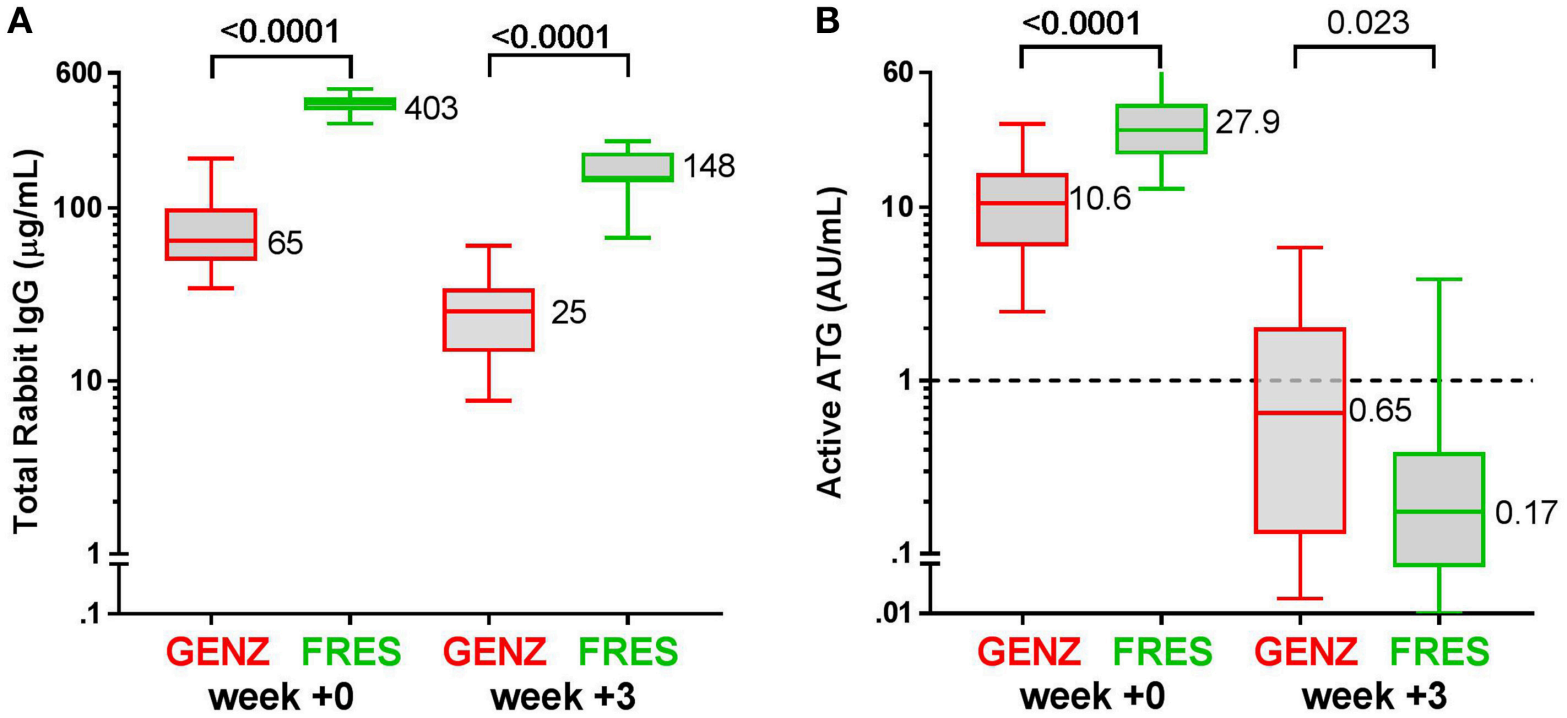
Box plots of serum/plasma concentration of total Rabbit IgG (total ATG) and active ATG in ATG-GENZ (Genzyme, red) and ATG-FRES (Fresenius, green) treated patients. **(A)** Total Rabbit IgG concentration in ATG-FRES treated patients at week 0 (day of graft infusion) and week +3 (expected time of neutrophil engraftment) is significantly higher in comparison with ATG-GENZ treated patients. **(B)** Active ATG concentration at week 0 in ATG-FRES treated patients is significantly higher in comparison with ATG-GENZ treated patients, but at week +3 the serum/plasma level of active ATG in ATG-FRES treated patients is lower than in ATG-GENZ treated patients (0.17 vs. 0.65 AU/mL).

In this study we aimed to compare clearance of the two brands of rabbit ATG, ATG-GENZ and ATG-FRES, given at different dosages and analyze the consecutive immune reconstitution in a homogeneous cohort of pediatric stem cell transplant recipients transplanted in two HSCT Centers (Table 1). First we compared the serum/plasma concentrations of rabbit IgG (total ATG) in 42 ATG-GENZ and 16 ATG-FRES treated patients during the first 3 months after HSCT. The concentration of total ATG-FRES at the day of HSCT was 5.8 times higher than that of ATG-GENZ (median total ATG in ATG-FRES group 403 μg/mL, range 306–485 μg/mL; in ATG-GENZ group 65 μg/mL, range 34–193 μg/mL; *p* < 0.0001, Figures 1A,B, 2A) and this corresponded with the higher total dose of ATG-FRES given (Table 1). Three weeks post-HSCT, around the expected time of engraftment, the rabbit IgG concentration was decreased similarly for both ATG brands, ATG-FRES from 403 to 148 μg/mL (factor 2.7), ATG-GENZ from 65 to 25 μg/mL (factor 2.6, Figure 2A), indicating that rabbit IgG from both brands was cleared at equal rates.

### Clearance of Active ATG

Subsequently, we determined the residual levels of T-cell binding ATG, i.e., active ATG. The active ATG concentration at the time of transplantation was 2.6 times higher for ATG-FRES than for ATG-GENZ (median active ATG in ATG-FRES group 27.9 AU/mL range 12.8–74.0 AU/mL; ATG-GENZ group median 10.6 AU/mL range 2.5–30.3 AU/mL, *p* < 0.0001, Figures 1C,D, 2B). However, 3 weeks post-HSCT, the active ATG concentration in patients receiving ATG-FRES had decreased by a factor 164 (27.9 to 0.17 AU/mL), while in patients receiving ATG-GENZ active ATG had decreased by a factor 16 (10.6 to 0.65 AU/mL (Figure 2B). Thus despite the higher starting levels of active ATG in ATG-FRES treated patients at transplantation, the rapid clearance actually resulted in lower levels of active ATG at 3 weeks post-HSCT, i.e., in only 1 of 16 patients (6%) this level was above 1 AU/mL whereas 17 out of 42 ATG-GENZ patients (40%) still had levels above 1 AU/mL at this time point (*p* = 0.012, Figures 1C,D).

### Active ATG Levels in Relation to Dosage

**Figure 3:**
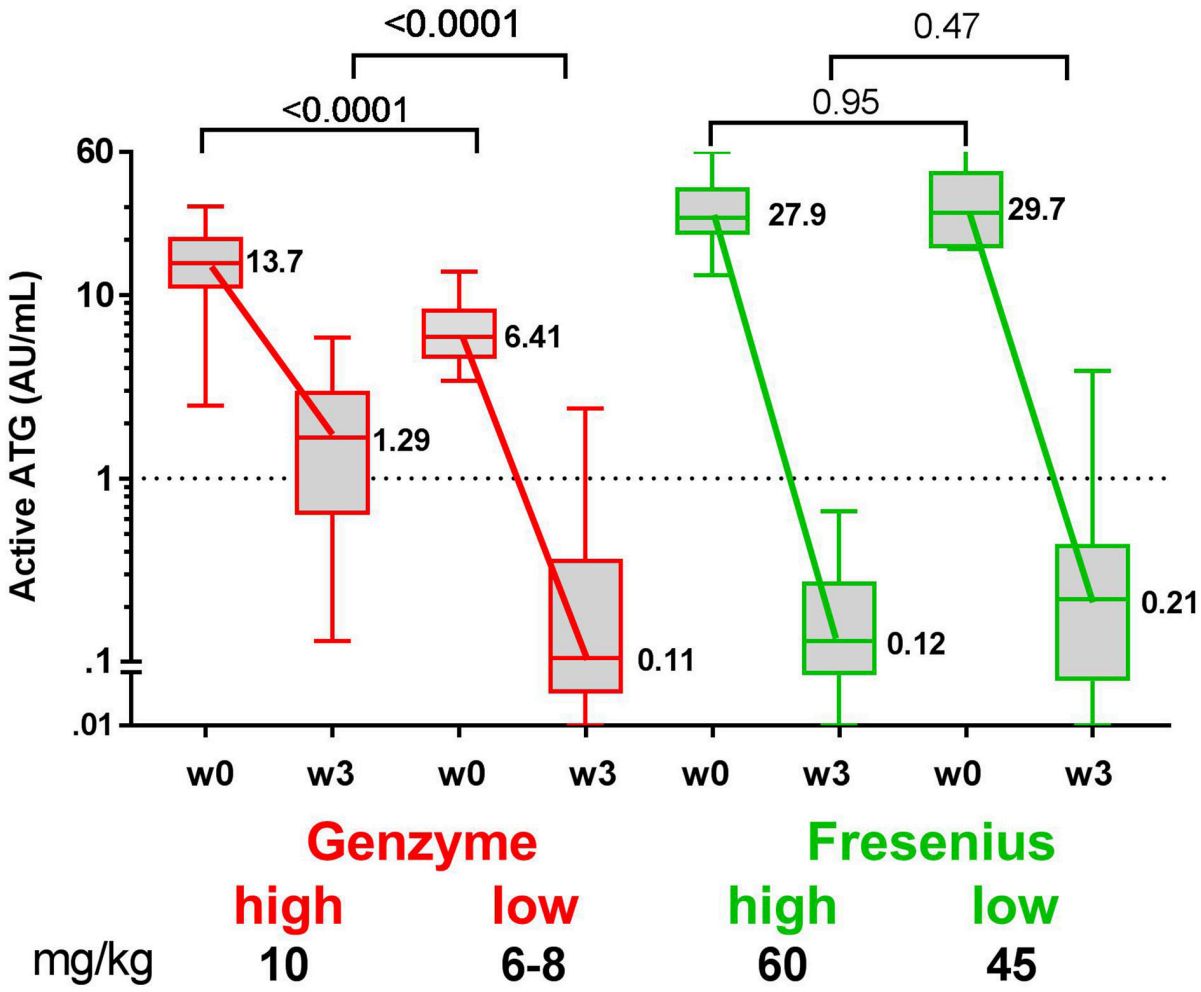
Box plots of serum/plasma concentration of active ATG in ATG-GENZ (Genzyme) and ATG-FRES (Fresenius) treated patients depending on the given total ATG dose. At week 0 (day of graft infusion) the ATG-GENZ patients receiving a total ATG dose of 10 mg/kg had a significantly higher serum/plasma level of active ATG than the 6–8 mg/kg receiving patients. Active ATG in ATG-FRES treated patients was at week 0 comparable between 45 and 60 mg/kg total ATG dose receiving patients and higher than in ATG-GENZ treated patients. At week +3 post-HSCT the serum/plasma level of active ATG in ATG-GENZ treated patients was still strongly dependent on the given dose (1.29 and 0.11 AU/mL), but the active ATG concentration in ATG-FRES treated patients was comparably low regardless of ATG dosing (0.12 and 0.21 AU/mL, respectively). The horizontal dashed line at 1 AU/mL is the highest serum/plasma concentration at which T-cells can recover.

Since in recent years the administered total doses of ATG were lowered for both brands, we investigated whether the dosage of ATG resulted in consistent differences in levels of active ATG post-HSCT and in the dynamics of immune cell reconstitution. We divided the ATG-GENZ group in patients receiving a total dosage of 10 mg/kg (ATG-GENZ-high; *n* = 24) and patients receiving a reduced dosage ranging between 6 and 8 mg/kg (ATG-GENZ-low; *n* = 18). The ATG-FRES group was divided in patients who received 60 mg/kg (ATG-FRES-high; *n* = 9) and patients receiving 45 mg/kg (FRES-low; *n* = 7) (Supplementary Table 1). The active ATG levels in ATG-FRES-high and ATG-FRES-low patients at the time of transplantation (median ATG-FRES-high 26.4 AU/mL vs. ATG-FRES-low 28.0 AU/mL; *p* = 0.95) and at three weeks post-HSCT (median ATG-FRES-high 0.13 AU/mL vs. ATG-FRES-low 0.22 AU/mL; *p* = 0.47, Figure 3) did not differ significantly. In contrast, patients receiving a high or low dosage of ATG-GENZ did differ significantly at the time of transplantation in the level of active ATG (ATG-GENZ-high 14.9 vs. ATG-GENZ-low 5.90 AU/mL; *p* < 0.0001) and at three weeks (1.67 AU/mL vs. 0.11 AU/mL; *p* < 0.0001, Figure 3). No relation between the number of lymphocytes pre-serotherapy and active ATG level at the day of transplantation was seen in this acute leukemia patient cohort (Supplementary Figure 2A). At three weeks post-HSCT 15 out of 24 ATG-GENZ-high patients (63%) had a level of active ATG above 1 AU/mL, while only 2 out of 18 (11%) ATG-GENZ-low patients still had an active ATG level above 1 AU/mL at this time point (*p* = 0.001). The number of total nucleated cells in the graft did not influence active ATG clearance (Supplementary Figures 2B,C).

### Immune Recovery in ATG-GENZ Subgroups and ATG-FRES Group

Patients in the ATG-FRES-high and ATG-FRES-low subgroups did not differ in active ATG levels at 3 weeks after HSCT (Figure 3) and their immune recovery was comparable: CD3+ T-cell recovery at 4 weeks, ATG-FRES-high 169 cells/μL, ATG-FRES-low 221 cells/μL, *p* = 0.6 (Supplementary Table 2). Therefore, data on immune recovery will be compared between the two ATG-GENZ subgroups and one combined ATG-FRES group.

**Figure 4:**
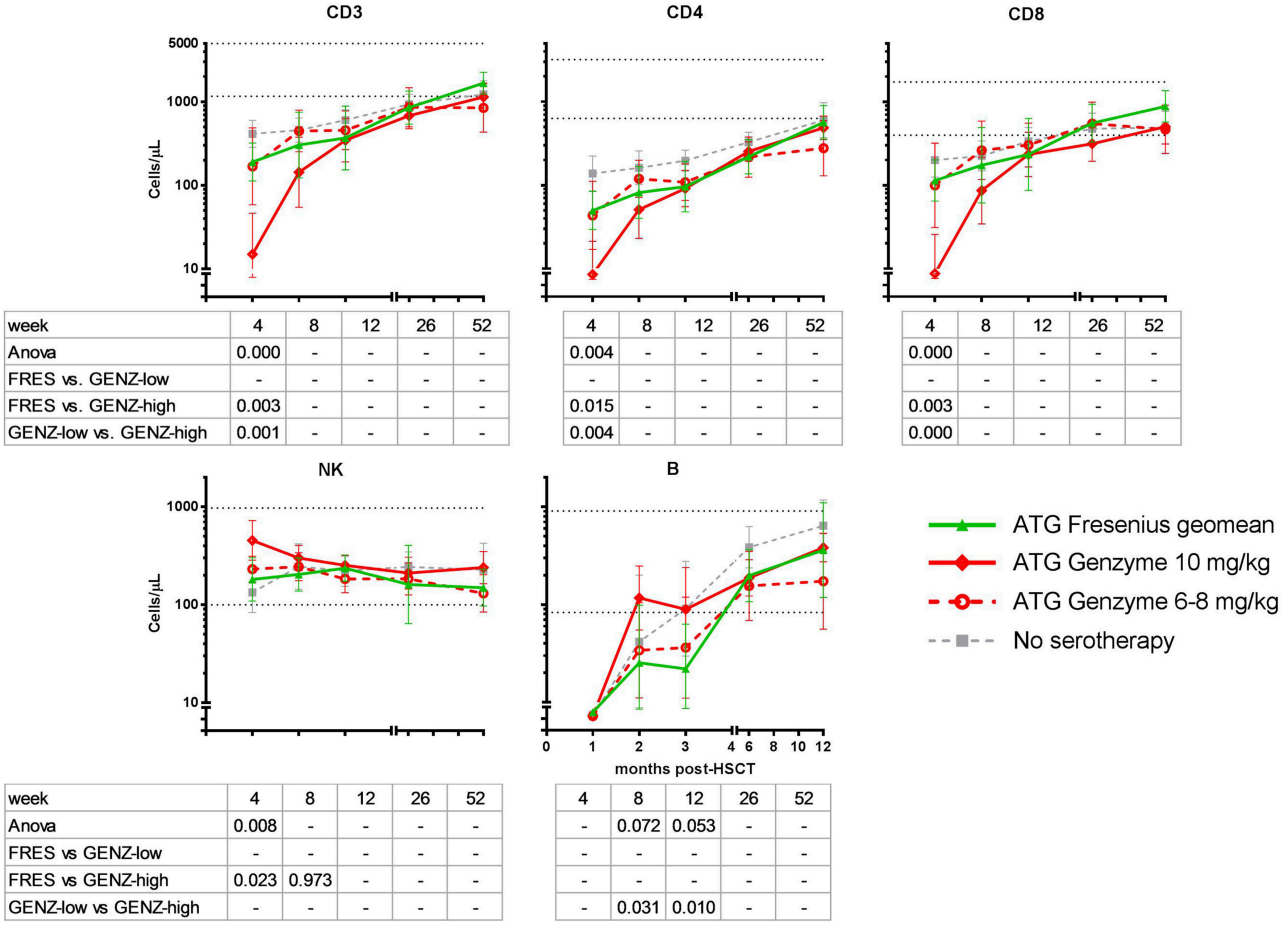
Immune cell recovery post-HSCT in ATG-FRES (Fresenius) treated patients in comparison with high (ATG-GENZ-high) and low dose (ATG-GENZ-low) ATG-GENZ (Genzyme) treated patients. CD3 T-cell and CD4 and CD8 T-cell subset numbers differed significantly at one month post-HSCT between the two ATG treated groups. ATG-GENZ-high patients recovered significantly slower for CD3, CD4, and CD8 compared to the ATG-FRES and ATG-GENZ-low treated patients. In contrast to the T-cell recovery, NK-cells recovered fast after HSCT reaching normal levels within 1 month. The highest level of NK-cell numbers were observed at one month in the ATG-GENZ-high group. B-cell recovery in all ATG groups returned to normal levels at 6 months post-HSCT with a significantly higher number of B-cells at 2 and 3 months in the ATG-GENZ-high compared to the ATG-GENZ-low and ATG-FRES treated patients, respectively. Dotted horizontal lines represent the 5th and 95th percentiles of cell numbers in 28 healthy age-matched donors. Gray line: patients transplanted for acute leukemia with a graft from an HLA-identical sibling donor without receiving ATG in the conditioning.

There was no difference in engraftment between the three groups, i.e., ATG-GENZ-high 22 days; ATG-GENZ-low 21 days and ATG-FRES 23 days; *p* = 0.86 (Table 2). However, T-cell (CD3+) numbers at 1 month post-HSCT were significantly lower in ATG-GENZ-high patients than in ATG-GENZ-low and ATG-FRES patients (Figure 4). T-cell numbers in these last two groups did not differ and were comparable to the numbers in the patients who did not receive serotherapy (ATG-GENZ-high: 15 cells/μL, ATG-GENZ-low: 165 cells/μL, ATG-FRES: 190 cells/μL; Anova *p* < 0.0001). A similar observation was made for the CD4+ and CD8+ T-cell subsets. The number of CD56+CD16± NK-cells at 1 month post-HSCT was significantly higher in ATG-GENZ-high patients (ATG-GENZ-high: 454 cells/μL, ATG-GENZ-low: 231 cells/μL, ATG-FRES: 182 cells/μL; Anova *p* = 0.008). The number of B-cells was very low for both brands at 1 month post-HSCT, but at 2 and 3 months post-HSCT ATG-GENZ-high patients had a significantly higher number of B-cells in comparison with ATG-GENZ-low (at 2 months *p* = 0.03; at 3 months; *p* = 0.01, Figure 4). No effect of TBI on immune recovery was observed in this study cohort (Supplementary Figure 3).

**Table 2:** Clinical outcome parameters in the ATG-Genzyme-high, ATG-Genzyme-low, and ATG-Fresenius group.

	**ATG Genzyme 10 mg/kg**	**ATG Genzyme 6–8 mg/kg**	**ATG Fresenius**	***p*-value**
Number of patients	24	18	16	
Engraftment failure, *n* (%)	0^*^	0	0	
Engraftment, day after HSCT median (range)	22 (13–46)	21 (12–30)	23 (14–27)	0.86
Acute GvHD, *n* (%)				0.024^†^
All grades	4 (17)	8 (44)	9 (56)	
Grade I	2 (8)	2 (11)	3 (19)	
Grade II	1 (4)	2 (11)	6 (38)	
Grade III–IV	1 (4)	4 (22)	0	0.025^†^
Chronic GvHD, *n* (%)				0.97
Limited	2 (8)	1 (6)	0	
Extended	4 (17)	2 (11)	2 (13)	
Viral infections, *n* (%) of patients at risk				
CMV	5 of 14 (36)	7 of 15 (47)	4 of 14 (29)^*^	0.62^†^
EBV	7 of 23 (30)	4 of 18 (22)	2 of 13 (15)^**^	0.28^†^
Relapse	4 (16)	4 (22)	3 (18)	0.54^†^
Transplant related mortality <100 days	1	0	0	
Overall survival, months median (range)	62 (1–92)	33 (4–53)	38 (4–84)	0.15^†^

*CMV, cytomegalovirus; EBV, Epstein-Barr virus; GvHD, Graft-vs.-Host Disease*.

* *One patient died (day +18) before engraftment*.

** *EBV status of three donor-patients couples pre-conditioning were lacking*.

† *Long rank test*.

### Clinical Outcome

**Figure 5:**
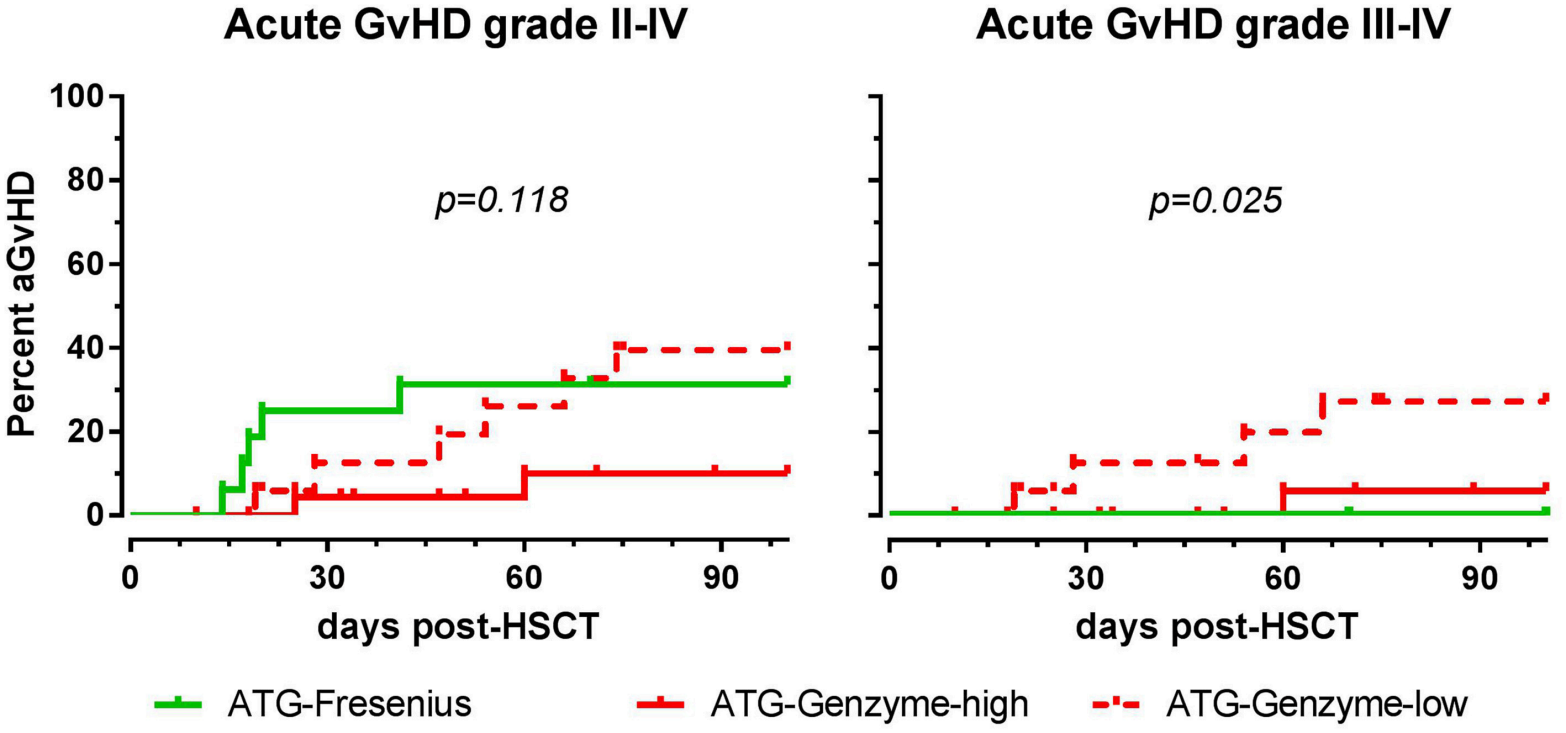
The effect of ATG brand and dosing on acute GvHD. Although not significant, acute GvHD (grade II-IV) occurred less frequently in ATG Genzyme high dose (ATG-GENZ-high) receiving patients compared to patients receiving a low dose of ATG-Genzyme (ATG-GENZ-low) or ATG Fresenius (ATG-FRES). For severe acute GvHD (grade III-IV), there was a significant difference between the three ATG groups. Severe acute GvHD occurred most frequently in the ATG-GENZ-low group.

The impact of differences in active ATG clearance and immune reconstitution on clinical outcome was investigated (Table 2). The overall incidence of acute GvHD (grade II-IV) tended to be lower in the ATG-GENZ-high subgroup (*p* = 0.118, Figure 5). GvHD grade III-IV did not occur in the ATG-FRES group, only in 1 out of 24 ATG-GENZ-high patients, and was significantly more frequent in ATG-GENZ-low patients (*p* = 0.025). The incidence of chronic GvHD, limited or extended, was comparable between the three groups (Table 2), and there was no difference in the incidence of relapse, CMV or EBV infection/reactivation and overall survival (OS, Supplementary Figure 4) between the three groups.

## Discussion

Studies on active ATG in HSCT patients have mainly been performed with ATG-Genzyme (7–9, 14, 15, 25, 34–36). Our current study is one of the few on ATG-Fresenius (16, 37) and the first one comparing the active components of the two rabbit ATG products.

While rabbit IgG (total ATG) of both products was cleared at similar rates (38), active ATG-FRES was cleared from the circulation faster than active ATG-GENZ, despite higher dosing of ATG-FRES and the presence of higher levels of active ATG at the day of transplantation. ATG-FRES was administered in two different dosages (the historical dosage of 60 mg/kg *vs*. the more recently given dosage of 45 mg/kg), but resulted in similar active ATG levels at 3 weeks after HSCT. The levels of active ATG in ATG-FRES treated patients were below the critical level for T-cell reconstitution of 1 AU/mL (15, 28) in all but one patient before 21 days post-HSCT. Thus, for ATG-FRES reducing the total dosage from 60 to 45 mg/kg has little impact on its *in vivo* persistence, probably because of the short half-life of its active component (16).

In contrast, there was a large variation in the day after HSCT when active ATG-GENZ fell below the level of 1 AU/mL, ranging from 0.5 to 7 weeks (14). Treatment with a lower ATG-GENZ dosage resulted in lower levels of active ATG at the day of HSCT as well as 3 weeks thereafter (9). For ATG-GENZ reducing the total dosage from 10 mg/kg to 6–8 mg/kg resulted in an increase from 11 to 63% of patients who had a level below 1 AU/mL at three weeks. This clearly had an impact on circulating T-cell numbers at 1 month after HSCT.

It is intriguing that active ATG from the two products is cleared at different rates. It can be assumed that this is due to differences in manufacturing, since ATG-FRES is absorbed to human placental cells and erythrocytes, while ATG-GENZ is absorbed to erythrocytes only (39). Another explanation might be the differences in antigens recognized by the polyclonal antibodies in both products. ATG-FRES is raised against the lymphoblastic Jurkat cell line. In contrast, ATG-GENZ is generated using human thymocytes expressing a different array of antigens as potential immunogens. As a result, the relative concentrations of antibodies recognizing various antigens expressed on T-cells vary between ATG-GENZ and ATG-FRES. For example, ATG-GENZ contains more antibodies recognizing CD8, CD11a, CD18, CD44, CD52, CD99 while ATG-FRES recognizes more CD5, CD98, and CD147 (5, 40), although other studies reported different antigens (41, 42).

ATG dose finding is pivotal in the delicate balance between T-cell elimination and post-HSCT immune reconstitution and has been the subject of a number of studies, although levels of active ATG were not always measured. For ATG-GENZ, a large variation in dosing has been reported, varying between 2.5 and 60 mg/kg (7, 10, 14, 43–50). In studies in which two dosages of ATG-GENZ were directly compared, the incidence of acute and/or chronic GvHD was lower in the group of patients who received a higher dose, as we observed in the present study (45, 47, 48, 51, 52). In contrast, when different dosages of ATG-FRES were compared in their efficiency of preventing GvHD, the differences between high and low doses were not obvious (27, 53, 54). The lack of an effect of the ATG-FRES dosage can be explained by our observation that active ATG-FRES is cleared rapidly and uniformly from the circulation. Likewise, reported data on T-cell reconstitution were less correlated with dose variation in ATG-FRES (45–60 mg/kg) in contrast to ATG-GENZ (6–10 mg/kg) (46, 48, 54). In retrospect, the reported effects of dosing of ATG-GENZ and lack of dosage effects in case of ATG-FRES may be explained by the differences in clearance of active ATG of both products.

Only a few studies have reported a comparison between the two brands of rabbit ATG with respect to T-cell reconstitution and clinical outcome after HSCT, however without taking clearance of the T-cell binding component (active ATG) into account (10, 17, 19) Although the number of patients in our study is limited, especially in the ATG-FRES group, none of the patients in the ATG-FRES group developed acute GvHD higher than grade II, whereas 22% of the patients in the ATG-GENZ-low group developed GvHD grade III or IV. Since time when active ATG levels fell below 1 AU/mL and T-cell reconstitution were comparable between these two groups, it would be interesting to investigate the recovery of functional T-cell subsets. Other studies have reported no difference in the occurrence of acute GvHD between patients receiving ATG-GENZ (range 5–10 mg/kg) and patients receiving ATG-FRES (range 20–60 mg/kg) (10, 17, 55). Protection from GvHD grade III-IV by ATG was reported before, but this was not limited to ATG-FRES (56).

From this retrospective study, we conclude that the two rabbit ATG products have different biopharmaceutical properties. During the last few years, physicians have empirically lowered the dosages of ATG in order to enhance T-cell reconstitution to prevent viral complications while maintaining sufficient immunosuppressive potential to prevent GvHD. Differences in dosing of ATG-FRES have little effect on the time when active ATG levels fall below 1 AU/mL and, therefore, are not expected to change functional T-cell reconstitution. However, reducing the dosing of ATG-GENZ results in less variable serum/plasma levels and, together with less variable clearance rates, this leads to a more predictable time frame at which the serum/plasma levels of active ATG decrease below the critical level of 1 AU/mL. Dosing of ATG-GENZ in the HSCT setting, therefore, appears more critical than dosing of ATG-FRES. However, in practice dosages of both products have to be further optimized, depending on the HSCT platform. Because of the observed differences in PK/PD of active ATG after administration of the two ATG brands, choices to use one or the other and the applied dosage should be carefully considered in clinical decision making.

## Author Contributions

LO did the research and wrote the manuscript. CJ-vdZ designed the study, analyzed the data, and wrote the manuscript. AJ-H did the research. KK, MI, and RB provided study material and clinical data and contributed to manuscript revision. AvH and MS wrote the manuscript. KM and AL established the collaboration and contributed to manuscript revision. MvT designed the study and wrote the manuscript.

### Conflict of Interest Statement

The authors declare that the research was conducted in the absence of any commercial or financial relationships that could be construed as a potential conflict of interest.

## Supplementary Material

Supplementary Figure 1 Active ATG Genzyme and Fresenius measurements on HUT-78 cells and T-cells in PBMC. **(A)** To validate the active ATG measurement the ATG-GENZ and ATG-FRES reference curves on HUT-78 cells were compared. The ATG-FRES curve is parallel shifted in comparison with the ATG-GENZ curve (factor difference = 2.6). These results confirmed the previously published observation that ATG-FRES contains less active ATG per μg rabbit IgG than ATG-GENZ(5). **(B)** The binding of active ATG (ATG-FRES or ATG-GENZ) in patients samples obtained at different time points after ATG infusion to HUT-78 cells was compared with the binding to the T-cell fraction in PBMC. For a wide range of concentrations of active ATG the results showed a good correlation for both products using either HUT-78 or T-cells in PBMC as target.

Supplementary Figure 2 The effect of lymphocytes pre start serotherapy and the total nucleated cell dose in the graft on the active ATG level. **(A)** No relation between the number of lymphocytes pre start serotherapy and active ATG level at the day of transplantation was observed in this acute leukemia patient cohort. **(B)** Patients were ordered based on the brand of ATG, the dose of ATG and the amount of nucleated cells in the graft, creating 6 different groups. The ATG-Genzyme high (10 mg/kg) and low (6–8 mg/kg) dosage treated patients getting a high number of nucleated cells, received a comparable number of nucleated cells as the Fresenius (both high 60 mg/kg and low 45 mg/kg) treated patients. **(C)** Factor of decrease of the active-ATG level at week 1 vs. 0 (day of transplantation) was highest in the Fresenius groups and was significantly different between the 4 ATG groups containing patients that received high numbers of nucleated cells (Kruskal-Wallis test: *p* = 0.0018). No significant difference in the decrease of this factor (ns, Genzyme 10 mg/kg group low vs. high NC: *p* = 0.536, Genzyme 6–8 mg/kg group low vs. high NC: *p* = 0.231) was observed in the ATG-Genzyme treated groups between patients that received a low or a high number of nucleated cells.

Supplementary Figure 3 The effect of TBI on T-cell recovery. No significant difference in T-cell recovery at 1, 2, and 3 months post-HSCT was observed between patients treated with or without TBI in the conditioning regimen. The Genzyme-low group was left out of this analysis since only one patient in this group received TBI. Figure shows geomeans and 95% confidence interval.

Supplementary Figure 4 The effect of ATG brand and dosing on clinical outcome parameters. No significant difference was observed between the ATG-Fresenius and the two ATG-Genzyme groups for CMV and EBV infection/reactivation (up to 6 months after HSCT), relapse of the acute leukemia or for overall survival (up to 5 years post-HSCT). For CMV and EBV only patients at risk (numbers in the table below the graph) were included in the analyses.
