# Supplementary figures and images for: Differential Elimination of Anti-Thymocyte Globulin of Fresenius and Genzyme Impacts T-Cell Reconstitution After Hematopoietic Stem Cell Transplantation
Source: Front Immunol. 2019 Mar 6;10:315. doi: 10.3389/fimmu.2019.00315 (PMC6414431; doi:10.3389/fimmu.2019.00315)

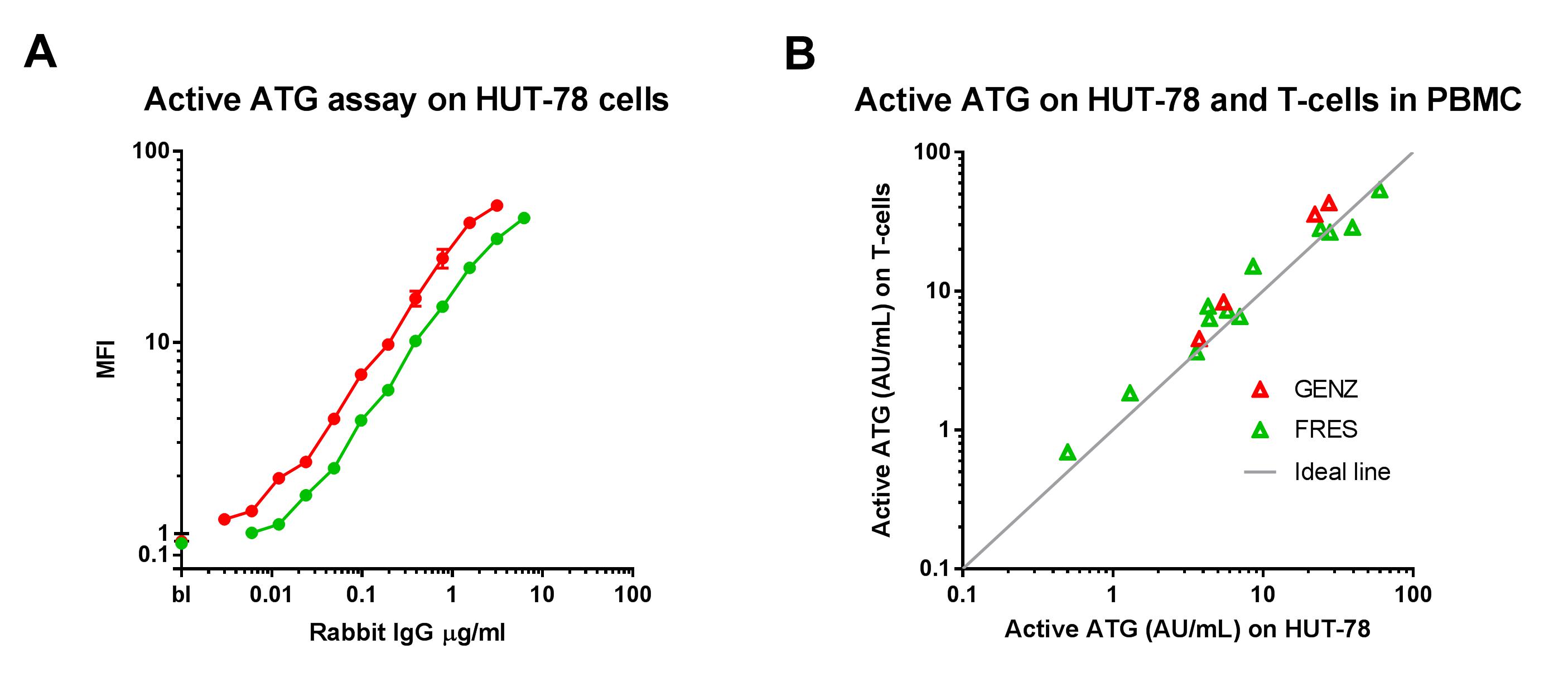

Supplement: Supplementary Figure 1 — Active ATG Genzyme and Fresenius measurements on HUT-78 cells and T-cells in PBMC. (A) To validate the active ATG measurement the ATG-GENZ and ATG-FRES reference curves on HUT-78 cells were compared. The ATG-FRES curve is parallel shifted in comparison with the ATG-GENZ curve (factor difference = 2.6). These results confirmed the previously published observation that ATG-FRES contains less active ATG per μg rabbit IgG than ATG-GENZ(5). (B) The binding of active ATG (ATG-FRES or ATG-GENZ) in patients samples obtained at different time points after ATG infusion to HUT-78 cells was compared with the binding to the T-cell fraction in PBMC. For a wide range of concentrations of active ATG the results showed a good correlation for both products using either HUT-78 or T-cells in PBMC as target. [file Image_1.JPEG]

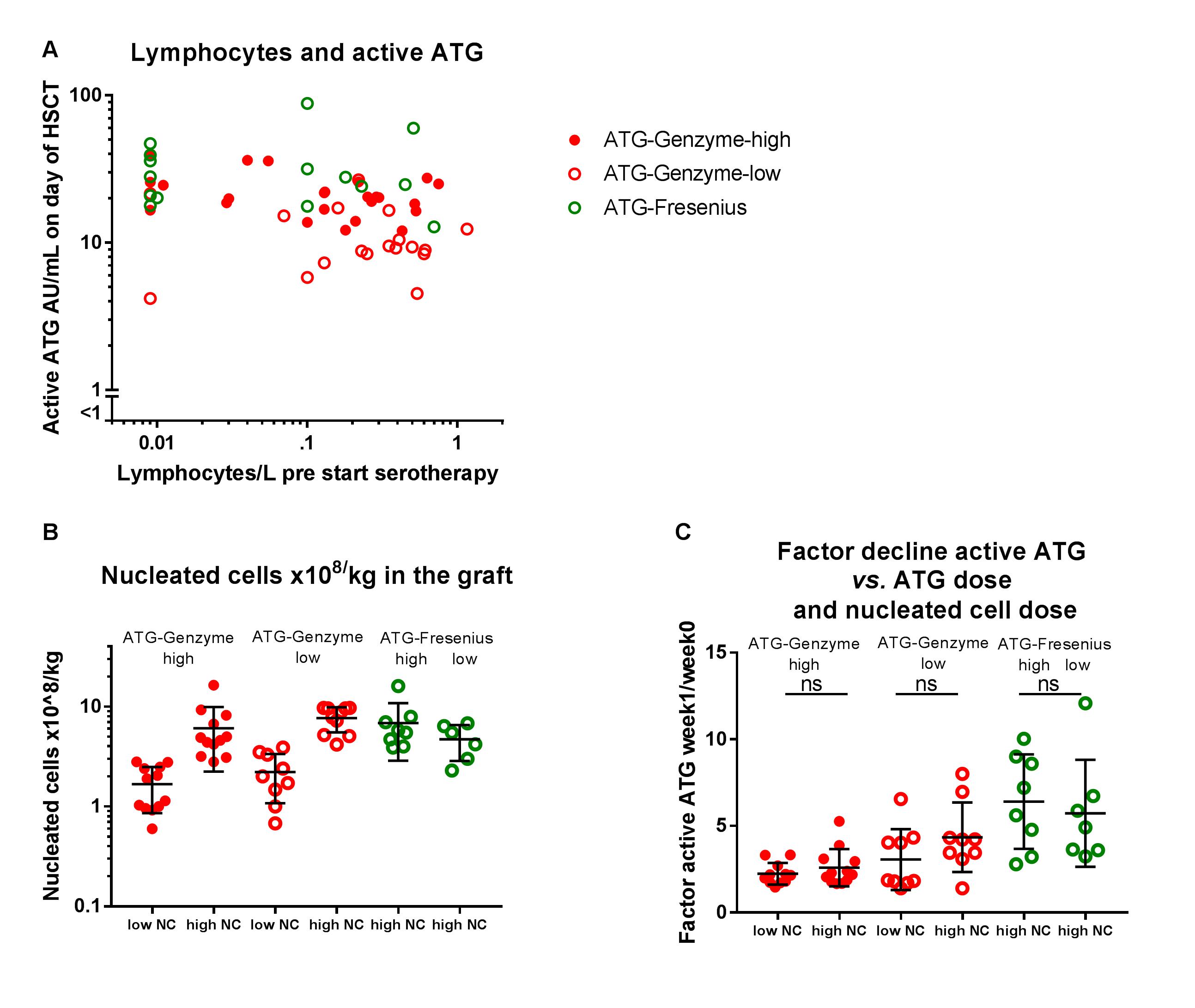

Supplement: Supplementary Figure 2 — The effect of lymphocytes pre start serotherapy and the total nucleated cell dose in the graft on the active ATG level. (A) No relation between the number of lymphocytes pre start serotherapy and active ATG level at the day of transplantation was observed in this acute leukemia patient cohort. (B) Patients were ordered based on the brand of ATG, the dose of ATG and the amount of nucleated cells in the graft, creating 6 different groups. The ATG-Genzyme high (10 mg/kg) and low (6–8 mg/kg) dosage treated patients getting a high number of nucleated cells, received a comparable number of nucleated cells as the Fresenius (both high 60 mg/kg and low 45 mg/kg) treated patients. (C) Factor of decrease of the active-ATG level at week 1 vs. 0 (day of transplantation) was highest in the Fresenius groups and was significantly different between the 4 ATG groups containing patients that received high numbers of nucleated cells (Kruskal-Wallis test: p = 0.0018). No significant difference in the decrease of this factor (ns, Genzyme 10 mg/kg group low vs. high NC: p = 0.536, Genzyme 6–8 mg/kg group low vs. high NC: p = 0.231) was observed in the ATG-Genzyme treated groups between patients that received a low or a high number of nucleated cells. [file Image_2.JPEG]

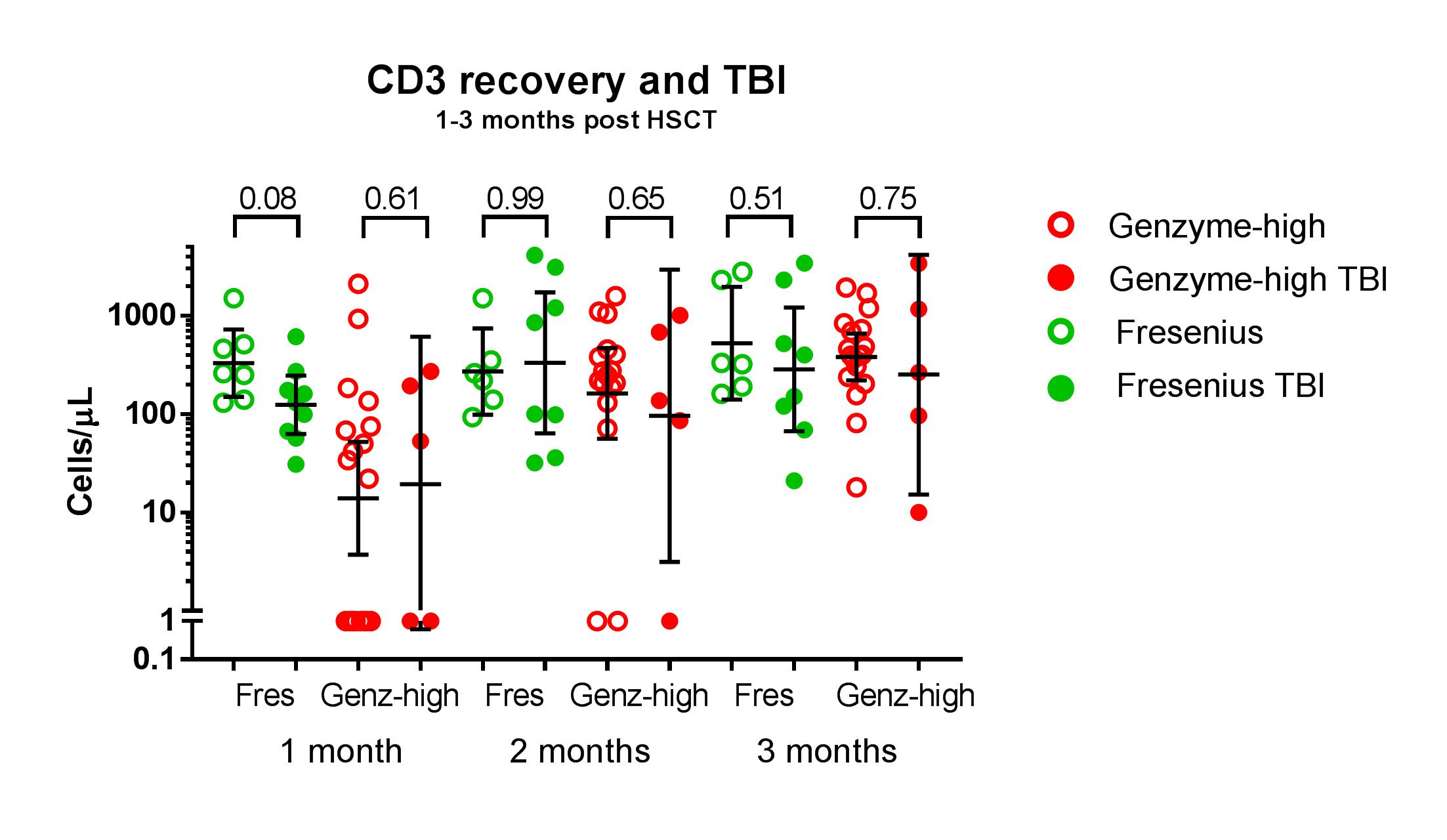

Supplement: Supplementary Figure 3 — The effect of TBI on T-cell recovery. No significant difference in T-cell recovery at 1, 2, and 3 months post-HSCT was observed between patients treated with or without TBI in the conditioning regimen. The Genzyme-low group was left out of this analysis since only one patient in this group received TBI. Figure shows geomeans and 95% confidence interval. [file Image_3.JPEG]

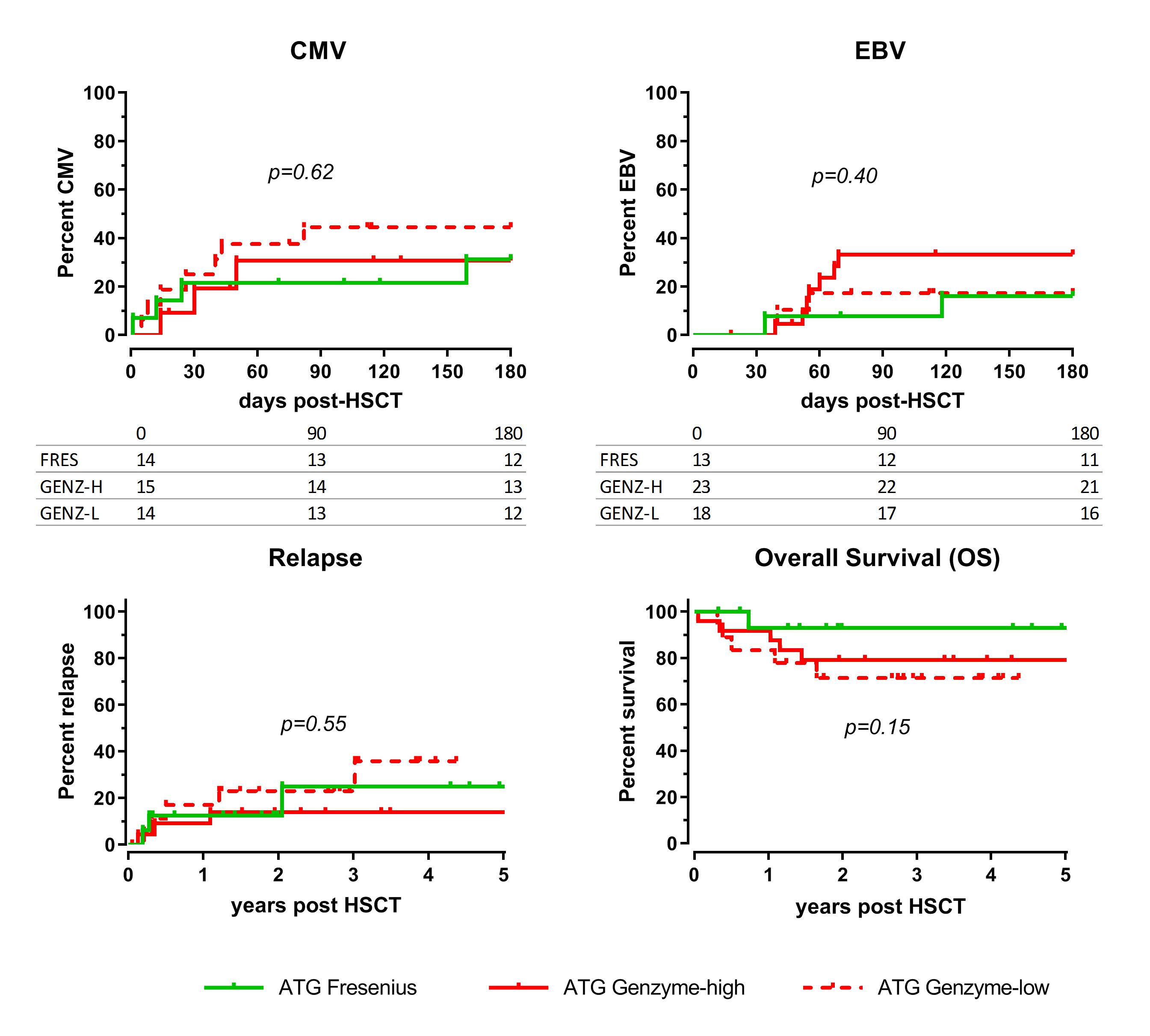

Supplement: Supplementary Figure 4 — The effect of ATG brand and dosing on clinical outcome parameters. No significant difference was observed between the ATG-Fresenius and the two ATG-Genzyme groups for CMV and EBV infection/reactivation (up to 6 months after HSCT), relapse of the acute leukemia or for overall survival (up to 5 years post-HSCT). For CMV and EBV only patients at risk (numbers in the table below the graph) were included in the analyses. [file Image_4.JPEG]
